# Genetic background-dependent effects of murine micro RNAs on circadian clock function

**DOI:** 10.1371/journal.pone.0176547

**Published:** 2017-04-27

**Authors:** Silke Kiessling, Ahmet Ucar, Kamal Chowdhury, Henrik Oster, Gregor Eichele

**Affiliations:** 1 Department of Genes and Behavior, Max Planck Institute for Biophysical Chemistry, Göttingen, Germany; 2 Nutrition and Immunology, Technical University of Munich, Freising, Germany; 3 Department of Molecular Cell Biology, Max Planck Institute for Biophysical Chemistry, Göttingen, Germany; 4 University of Manchester, Faculty of Biology, Medicine and Health, Division of Clinical and Molecular Cancer Sciences, Manchester, United Kingdom; 5 Chronophysiology Group, Medical Department I, University of Lübeck, Lübeck, Germany; University of Texas Southwestern Medical Center, UNITED STATES

## Abstract

MicroRNAs (*miR*s) are important regulators of a wide range of biological processes. Antagomir studies suggest an implication of *miR-132* in the functionality of the mammalian circadian clock. *miR-212* and *miR-132* are tandemly processed from the same transcript and share the same seed region. We found the clock modulator *miR-132* and *miR-212* to be expressed rhythmically in the central circadian clock. Consequently, mRNAs implicated in circadian functions may likely be targeted by both *miRs*. To further characterize the circadian role we generated mice with stable deletion of the *miR-132/212* locus and compared the circadian behavior of mutant and wild-type control animals on two genetic backgrounds frequently used in chronobiological research, C57BL/6N and 129/Sv. Surprisingly, the wheel-running activity phenotype of *miR* mutant mice was highly background specific. A prolonged circadian free-running period in constant darkness was found in 129/Sv, but not in C57BL/6N *miR-132/212* knockout mice. In contrast, in C57BL/6N, but not in 129/Sv *miRNA-132/212* knockout mice a lengthened free-running period was observed in constant light conditions. Furthermore, *miR-132/212* knockout mice on 129/Sv background exhibited enhanced photic phase shifts of locomotor activity accompanied by reduced light induction of *Period* gene transcription in the SCN. This phenotype was absent in *miRNA-132/212* knockout mice on a C57BL/6N background. Together, our results reveal a strain and light regimen-specific function of *miR-132/212* in the circadian clock machinery suggesting that *miR-132* and *miR-212* act as background-dependent circadian rhythm modulators.

## Introduction

24-hour, or circadian, clocks have evolved as an adaptation to daily recurring changes in the environment. In mammals, the central circadian pacemaker is localized in the suprachiasmatic nucleus (SCN) of the ventral hypothalamus [1, 2]. Generation of circadian rhythmicity occurs at the single cell level and is based on interlocked positive and negative transcriptional-translational feedback loops (TTL) of clock genes and their protein products [3].

Light is the major external stimulus that entrains circadian rhythms to the external 24-h light/dark cycle. External light information is perceived by retinal ganglion cells and transmitted through the retino-hypothalamic tract to target neurons in the SCN [4]. In the SCN, photic stimuli are transduced to the molecular clockwork by several signaling pathways (reviewed in [5]) and directly evoke expression of the clock genes *Period 1* and *Period 2* (*Per1/2*) [6].

In any cell type, up to 20% of the transcriptome is under circadian control [7]. By this mechanism circadian clocks regulate major cellular and physiological processes, such as energy metabolism [8]. Circadian clock oscillations are regulated at various levels, ranging from transcription and translation to subcellular localization and targeted degradation of clock proteins. Clock proteins appear several hours after their cognate mRNA transcripts [9], suggesting that their translation is tightly regulated, probably including *microRNA* (*miR*) -mediated mechanisms [10]. *miR*s are small (~22-nt) noncoding RNAs which regulate gene expression by influencing the translation of their target mRNAs [11]. Expression of 30% of mammalian genes is regulated by *miR*s [12] and about 13% of murine hepatic *miR*s were found to be expressed in a time-of-day-dependent manner [13].

Transcriptome analyses revealed marked variations in the expression level of genes in different mouse strains [14, 15]. Mouse strain-specific differences of *miR* abundance and function were documented in the brain and peripheral tissues for various *miR* species [16–18]. For example, RT-PCR screens reported expression differences in 11 out of 166 *miR*s among four inbred mouse strains, A/J, BALB/cJ, C57BL/6J, and DBA/2J [17]. *miR*s are believed to act as modifiers, for example on disease-causing genes [19], and accumulating evidence suggests that *miR*s are significant players in regulating various aspects of circadian clock function. For example, *miR-192/194* down-regulate *Per* gene expression *in vitro* leading to a shortening of the circadian period length [20]. Expression of *Aryl hydrocarbon receptor nuclear translocator-like* (*Arntl* or *Bmal1*) is down-regulated by *miR-494*, *miR-152* and *miR-142/-143* in mice [21]. *miR-182* regulates *Circadian locomotor output cycles kaput* (*Clock*) gene expression in rats by targeting its untranslated region (UTR) [22]. Recently, the rhythmically expressed *miR-17-5p* was found to shorten free-running period length by targeting the UTR of *Clock* mRNA and *Nuclear PAS domain protein 2* (*Npas2*) expression in the SCN [23].

In the present study, we investigate the circadian function of *miR-132* and *miR-212* in two mouse strains frequently used in chronobiological research. *miR-132* and *miR-212* are produced from a single transcript encoded by the murine *miR-132/212* locus on chromosome 11 [24]. Both share predicted target genes (http://www.microrna.org/; http://www.pictar.org/), which are known modifiers of the circadian clock and expressed in the SCN, such as *Mitogen-activated protein kinase 8* (*Mapk8*) or *Sirtuin 1* (*Sirt1*) [25, 26]. Due to their identical “seed” sequence [27] and in order to avoid redundancy effects after targeting of either *miR-132* or *miR-212* alone, we have used mice lacking both *miR*s (*miR-132*^*-/-*^*/212*^*-/-*^). Antagomir application had previously identified *miR-132* as modulator of light-induced clock gene expression in the SCN and activity resetting [28]. This role for *miR-132* as modulator of clock phase shifting was supported by overexpression experiments [29]. However, while overexpression of *miR-132* suppressed light-induced *Per1* expression consistent with reduced behavioral phase shifts, down regulation of *miR-132* by antagomir treatment also caused reduced light induced PER2 expression, in apparent contradiction to an enhanced behavioral phase shifting [28, 29]. Such apparent discrepancies between studies may be caused, at least in part, by genetic background effects on *miR-132* function. This prompted us to directly examine whether genetic alterations between different mouse strains contribute to the phenotypic circadian variability in *miR* mutants. We found that knockout of *miR-132/212* affects SCN period length and photic resetting differentially in C57BL/6N and 129S2/SvPasOrlRj (129/Sv) inbred mouse strains.

## Materials and methods

### Ethical statement

All animal experiments were done with prior ethical assessment by and permission from the Office of Consumer Protection and Food Safety of the State of Lower Saxony and in accordance with the German Law of Animal Welfare.

### Study design

Animals were kept for 2 weeks under LD conditions ("lights on", *zeitgeber* time (ZT) 0; "lights off", ZT12; light intensities of 100 lux) and then released into constant darkness (DD) for at least 14 days (DD_1_) to compare the free-running period length. To analyze the resetting ability an activity-delaying 15-min light pulse (LP) of 20 lux was given at circadian time (CT) 15 (3 hours after activity onset) on day 15 during DD_1,_ followed by another period of darkness for 14 days (DD_2_) and subsequent release into constant light (LL) of 100 lux to compare the free-running period in LL. The color of the fluorescent light bulbs used during all experiment was around 3k Kelvin. Wheel-running activity including behavioral phase shifts were analyzed using ClockLab software (Actimetrics, Wilmette, IL, USA).

### Experimental animals

For all experiments, 2–3 months old in-house bred WT and homozygous *miR-132*^*-/-*^*/212*^*-/-*^ (KO) littermate mice in either of the background strains (C57BL/6N and 129/Sv) were used The miR-212/132 null mouse line has been generated by genomic targeting of the *miR-212/132* locus in ES cells that has been derived from inbred wild-type 129/Sv (full name: 129S2/SvPasOrlRj) background. Therefore the mouse line that has been obtained by breeding the founder mice with 129/SV wild-type animals were in pure 129/Sv background, and this line has been maintained at heterozygous state. The founder mice were also bred in parallel with the wildtype C57BL/6N mice and the heterozygote progenies has been subsequently bred with the wildtype C57BL/6N mice for a minimum of 6 generations to obtain miR-212/132 null mouse line in almost pure (>%98, N6) C57BL/6N background. For both mouse lines in 129/Sv and C57BL/6N backgrounds, we have generated the homozygous null mice for analyses via breeding heterozygous animals with each other and have used the wildtype and homozygous mutant littermates in pairs. KO mice were viable in, either, a *129/Sv* or a *C57BL/6N* background and did not show any obvious alterations in behavior, although involvement of both *miR*s had previously been described for hematopoietic cell development and for endothelial and cardiac functions [30–32]. Thus, it was feasible to compare running-wheel performance as a sensitive readout of central circadian clock function in both KO mouse lines. Only male mice were used to avoid additional rhythmic components in females, such as the estrous cycle, which may influence general activity and wheel-running performance [33]. Before experiments mice were bred and housed in small groups of 5 or fewer in the same animal facility under the same LD cycle conditions of 12 hours light, 12 hours dark (LD), with food and water *ad libitum*. Throughout the experiments animals were kept under constant temperature (20±0.5°C) and humidity (50–60%).

### Behavioral analysis

Handling and activity measurements during experiments were performed as described previously [34]. For constant light conditions and light pulse experiments, animals were single-housed in running wheel-equipped cages (33 x 15.5 x 13 cm with a 15-cm diameter wheel) with standard bedding. At least 10 days of wheel-running recordings for each condition were used to determine activity period (tau, calculated using a χ^2^ periodogram and confirmed by fitting a line to the onsets of activity), phase shifts (difference between regression lines through the onsets of activity before and after the light pulse) and the total amount of activity. Activity profiles have been calculated with the ClockLab software by averaging the data from 10 consecutive days of wheel running activity in each light conditions. Wheel rotations were detected in 6 min intervals and calculated either on a 24 h day or relative to the individual day length of each mouse based on their individual free-running period in DD and LL conditions. The group sizes were as follows: LD profile 129/Sv WT n = 9; KO n = 6; C57BL/6N WT n = 11; KO n = 10; DD profile 129/Sv WT n = 8; KO n = 6; C57BL/6N WT n = 11; KO n = 10; LL profile: 129/Sv WT n = 5; KO n = 6; C57BL/6N WT n = 11; KO n = 10; total activity in DD 129/Sv WT n = 7; KO n = 8; C57BL/6N WT n = 8; KO n = 6; total activity in LL 129/Sv WT n = 5; KO n = 6; C57BL/6N WT n = 10; KO n = 10; period in DD 129/Sv WT n = 15; KO n = 15; C57BL/6N WT n = 12; KO n = 11; period in LL 129/Sv WT n = 6; KO n = 7; C57BL/6N WT n = 10; KO n = 11; phase shift 129/Sv WT n = 8; KO n = 9; C57BL/6N WT n = 11; KO n = 10.

### In situ hybridization (ISH)

Circadian profiles of *miR-132* and *miR-212* expression were determined in the SCN of 129/Sv wildtype mice. Brains were dissected at 6 CTs (CT3, 7, 11, 15, 19, 23) during the second day in DD (n = 18 mice; 3 mice per time point) following 2 weeks of LD. Digoxigenin-labelled ISH was performed in 129/Sv and C57BL/6N wildtype mice as previously reported [35] for *Per1* and *Per2* and signal strength quantified as has been described [36]. For each individual animal, 3–6 sections spanning throughout the SCN were evaluated and a background-subtracted mean value calculated. Mean SCN expression of each *miR* is represented relative to the averaged expression over the circadian day (set to 1). To measure the induction of gene expression after a light pulse at CT 15 (15 min, 100 lux) (n = 4 per time point and genotype) animals were sacrificed 1 hour later by cervical dislocation (under dim red light during the dark period) and brains were dissected. Tissues were fixed, dehydrated, and paraffin embedded; 8-μm sections were prepared and stored at –80°C. Sections were hybridized with ^35^S-UTP-labelled antisense RNA probes for clock gene transcripts [37]. Relative quantification of expression levels was performed by densitometric analysis of autoradiograph films using Scion Image software (Scion Corp. Frederick, MD, USA).

### Statistical analysis

All statistics were performed using GraphPad Prism 5.0 (Prism, La Jolla, CA, USA) software. Diurnal variation of *miR-132* and *miR-212* expression data was tested by fitting a cosine wave (equation y = B + (A * cos (2 * *π* * ((x–Ps) / 24)))) to gene expression data, where B is the baseline, A is the amplitude, Ps is the phase shift, with a fixed 24-hour period; significance was determined by F-test. Two-way ANOVA was performed to detect statistical differences of period length, light pulse period length effects, light-induced phase shifts and gene induction followed by Sidak's multiple comparison post-hoc tests. Paired t-test was used to determine period variability between groups and within animals by comparing the period length in DD before and after a light pulse. Correlation analysis of period length and phase shift was performed by fitting a linear regression. The corresponding p-value was determined using the Pearson correlation coefficient, given the correlation value and the sample size. A statistically significant difference was assumed with p-values of less than 0.05. Asterisks indicate significance (* p ≤ 0.05; ** p ≤ 0.01; *** p < 0.001).

## Results

### miR-132/212-deficiency differentially affects the free-running period length of locomotor activity in 129/Sv and C57BL background strains

**Fig 1:**
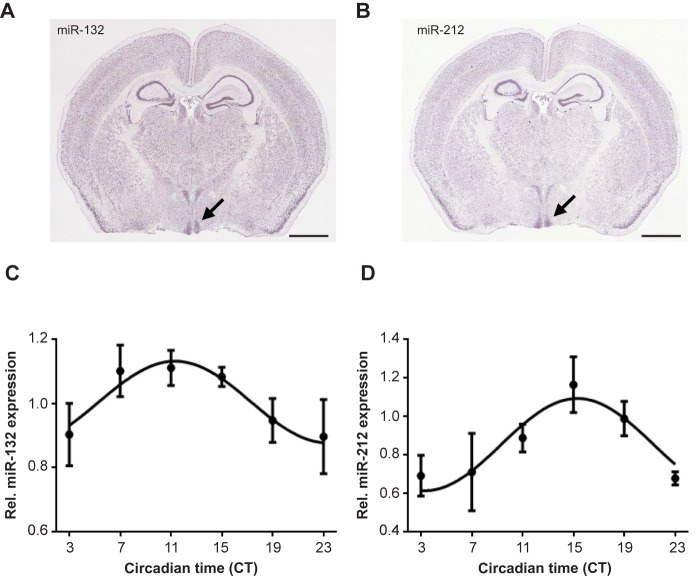
miR-132 and miR-212 expression in the SCN. (A, B) Gene expression patterns of *miR-132* (A) and *miR-212* (B) in the brain of 129/Sv mice measured by ISH. SCNs are indicated by arrows. (C, D) Relative gene expression profiles in constant darkness of miR-132 (C) and miR-212 (D) in the SCN of 129/Sv mice measured by dig-labelled ISH. Significant rhythms are illustrated with fitted cosine curves (Cosine-wave regression, F-test: (C) *miR-132*: p < 0.01, (D) *miR-212*: p < 0.05). Scale bar in A and B: 2 mm.

**Fig 2:**
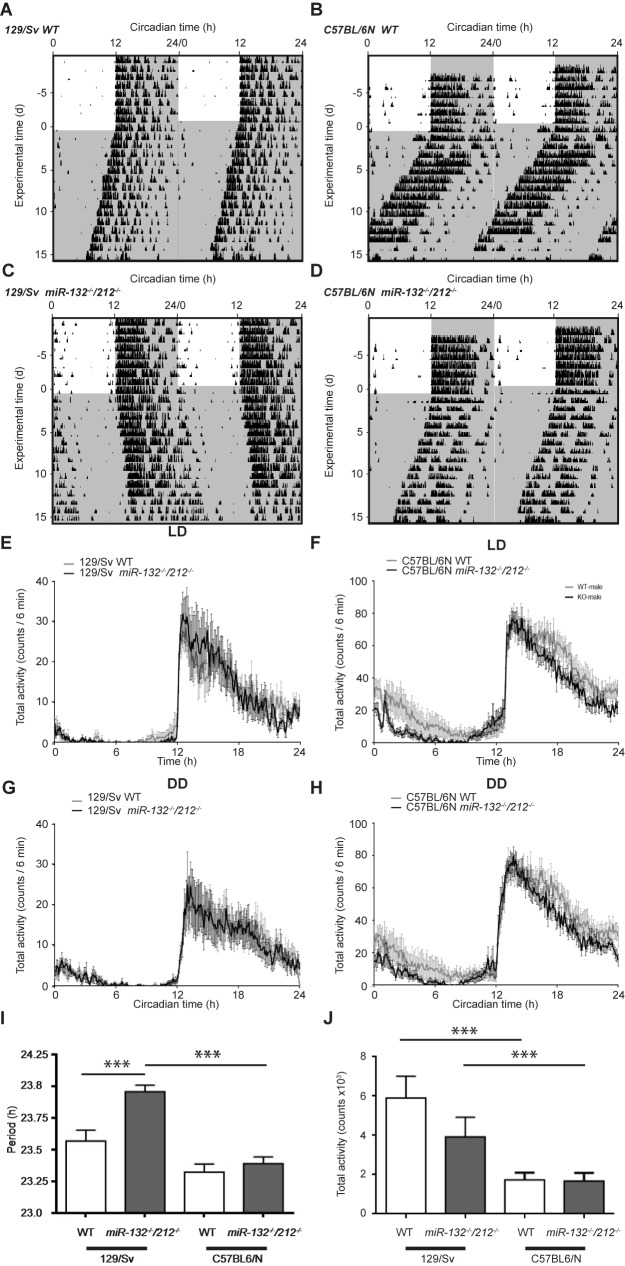
Influence of miR-132/212 deletion on the period length in constant darkness. (A-D) Representative double-plotted actograms of WT (A, B) and KO (C, D) mice in LD and DD. (E-H) Total activity profiles of WT and KO mice during LD (E, F) and DD (G, H) conditions in 129/Sv (E, G) and C57BL/6N backgrounds (F, H). (I, J) Period length (I) and total activity (J) in constant darkness for all genotypes. Period length: 129/Sv: WT: 23.57 ± 0.08 h, n = 15; KO: 23.96 ± 0.05 h, n = 15; C57BL/6N: WT: 23.32 ± 0.07 h, n = 12; KO: 23.39 ± 0.05, n = 11, two-way ANOVA for interaction F(1, 49) = 5.390, p = 0.0244, Sidak’s post-hoc test for background difference: WT: p > 0.05; KO, p < 0.001, Sidak’s post-hoc test for genotype difference 129/Sv: p < 0.001, C57BL/6N: p > 0.05. Total activity: 129/Sv: WT: 5904 ± 1104 counts, n = 7; KO: 3,921 ± 1,003 counts, n = 8; C57BL/6N: WT: 1,729 ± 376 counts, n = 8; KO: 1679 ± 408, n = 6, two-way ANOVA for genotype F(1, 31) = 1.358, p = 0.2527, two-way ANOVA for background F(1, 31) = 51.34, p < 0.001. Data are represented as mean ± SEM., * p < 0.05, ** p < 0.01, *** p < 0.001.

*miR-212* and *miR-132* are strongly expressed in the SCN of 129/Sv mice (Fig 1A and 1B) and exhibit rhythmic transcription profiles under constant darkness (DD) conditions (Fig 1C and 1D). Both *miRs* share the same seed sequence and thus may target the same mRNAs. Indeed, the main functions of *miR-212* and *miR-132* overlap in the context of neuronal or immune functions. Thus, to avoid possible redundancy, we have examined the circadian role of the *miR-132/212* family in double knockout mice. To characterize SCN pacemaker function under free-running DD conditions, running-wheel activity of *miR-132*^*-/-*^*/212*^*-/-*^ mice on either genetic background was compared against their respective wild-type (WT) littermates. Period length in WT mice varied slightly, but not significantly, between the two background strains, whereas the different KO lines showed a significant difference of about 34 min in their free-running period length (Fig 2A–2D and 2I). *MiR-132/212* loss-of-function in the 129/Sv background resulted in a moderate, but statistically significant increase of the circadian period by about 24 min (Fig 2A, 2C and 2I). In contrast, no significant effect on free-running period length was observed in *miR-132*^*-/-*^*/212*^*-/-*^ mice in C57BL/6N background (Fig 2B, 2D and 2I). Total activity in mice of both genotypes on 129/Sv background (Fig 2E, 2G and 2J) was higher compared to C57BL/6N (Fig 2F, 2H and 2J). However, total activity within each background strain under LD and DD conditions was indistinguishable between WT and KO mice (Fig 2E–2H).

**Fig 3:**
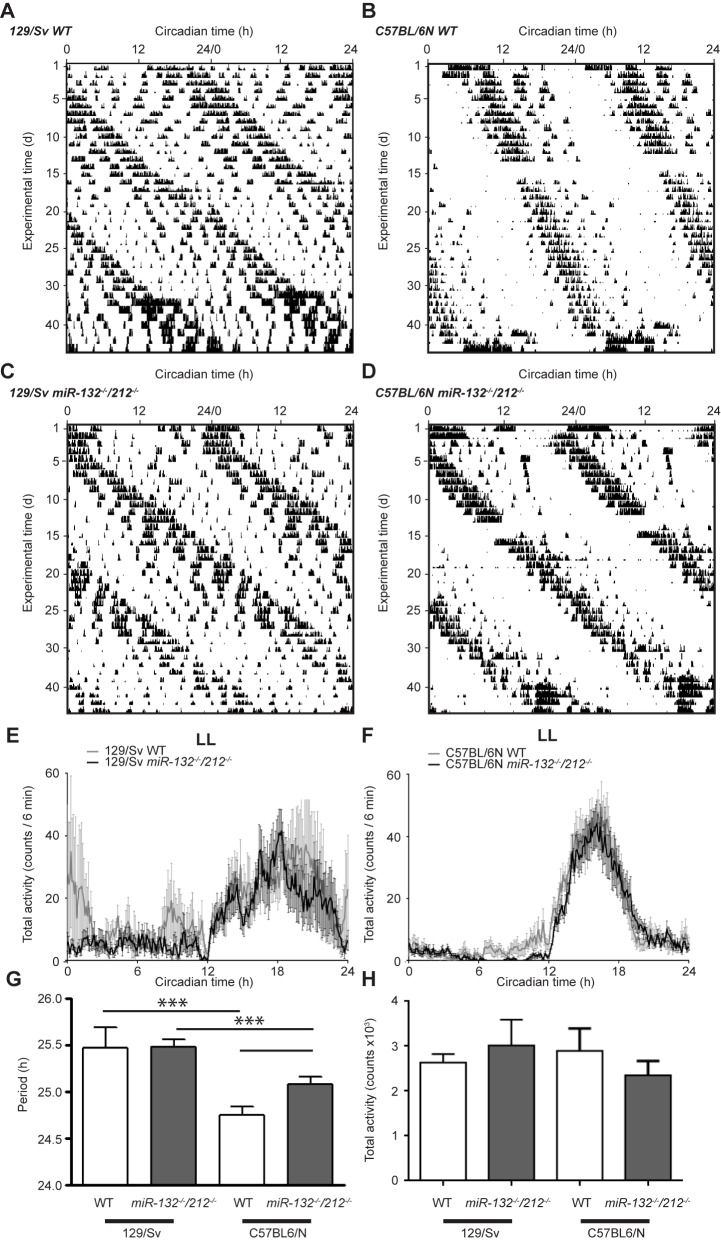
Influence of miR-132/212 deletion on the period length in constant light. (A-D) Representative double-plotted actograms of WT (A, B) and *miR-132*^*-/-*^*/212*^*-/-*^ (C, D) mice in constant light conditions. (E, F) Total activity profiles of WT and KO mice in 129/Sv (E) and C57BL/6N backgrounds (F). (G, H) Period length (G) and total activity (H) in constant light of 100 lx for all genotypes. Period length: 129/Sv: WT: 25.47 ± 0.22 h, n = 6; KO: 25.49 ± 0.08 h, n = 7; C57BL/6N: WT: 24.71 ± 0.08 h, n = 10; KO: 25.08 ± 0.08 h, p = 0.0143, n = 11, two-way ANOVA for interaction: F(1, 29) = 4.341, p = 0.0461, Sidak’s post-hoc test for background differences: p < 0.001, Sidak’s post-hoc test for genotype differences 129/Sv: p > 0.05, C57BL/6N: p < 0.05. Total activity: 129/Sv: WT: 2,636 ± 190 counts, n = 5; KO: 3,016 ± 576 counts, n = 6; C57BL/6N: WT: 2,895 ± 499 counts, n = 10; KO: 2,352 ± 319, n = 10,; two-way ANOVA for interaction F(1, 27) = 0.9622, p = 0.3353, two-way ANOVA for background F(1, 27) = 0.185, p = 0.6705, two-way ANOVA for genotype F(1, 27) = 0.02991, p = 0.8640. Data are represented as mean ± SEM., * p < 0.05.

Constant light conditions (LL; 100 lux) increased the period length in both genotypes on 129/Sv (Fig 3A, 3C and 3G) and C57BL/6N (Fig 3B, 3D and 3G) background, consistent with previous reports [38]. Strain dependent alterations in activity profiles were apparent (compare Fig 3E and 3F), but total activity in LL was indistinguishable between the cohorts of both background strains and genotypes (Fig 3H). Similar to results obtained in DD conditions, in LL, WT mice showed background strain dependent differences in period length (Fig 3A–3D and 3G). Loss of *miR-132/212* did not affect LL period length in 129/Sv mice (Fig 3A, 3C and 3G), but increased the circadian day length in mice on a C57BL/6N background (Fig 3A–3D and 3G). Total activity in LL was altered neither by genetic background nor genotype (Fig 3H).

Taken together, *miR-132/212*-deficiency lengthened the circadian period of the SCN pacemaker in a strain- and light regimen-dependent way. Loss of *miR-132/212* prolonged the free-running period in constant darkness in the 129/Sv background, while increasing the free-running period in constant light conditions in the C57BL/6N strain.

### miR-132/212-deficiency differentially affects photic phase shifts in C57BL/6N and 129/Sv strains

**Fig 4:**
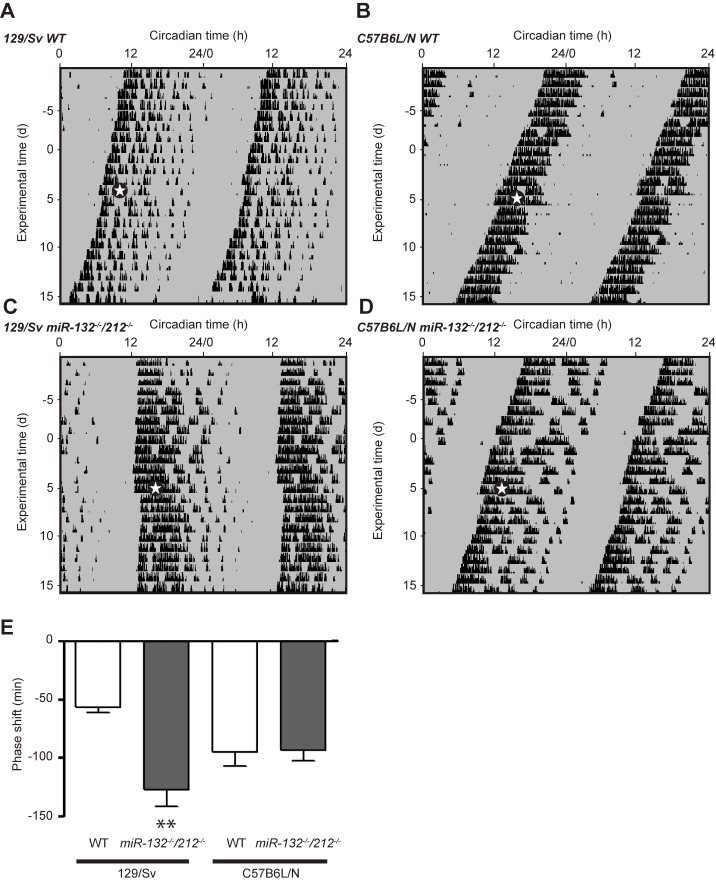
Influence of miR-132/212 deletion on behavioral phase shifts after single light pulses. (A-D) Representative actograms of WT (A, B) and KO (C, D) mice in constant darkness showing a brief (15-min) light pulse at CT15 of 20 lx which is marked by white stars and separates the free-running phase in two darkness periods (pre and post shift). (E) Magnitudes of phase shifts. 129/Sv: WT: -57.14 ± 4.5 min, n = 8; KO: -114 ± 15.4 min, n = 9; C57BL/6N: WT: -95 ± 12.1 min counts, n = 11; KO: -93.8 ± 8.4 min, n = 10,; two-way ANOVA for interaction F(1, 34) = 6.530, p = 0.0152, Sidak’s post-hoc test for genotype 129/Sv: p < 0.01, C57BL/6N: p > 0.05. Data are represented as mean ± SEM., ** p < 0.01.

Changes in the circadian period length as described above tend to correlate with alterations in the light resetting capability of the SCN pacemaker [39]. To address this in *miR-132*^*-/-*^*/212*^*-/-*^ mouse lines more directly, we measured acute phase-shifting of the wheel-running activity rhythm in response to a delaying 15-min light pulse (20 lux) at CT15 on 129/Sv (Fig 4A and 4C) and C57BL/6N background (Fig 4B and 4D; of note, these were the same cohorts of C57BL/6N animals used for the experiments shown in Fig 2A–2D). Indeed, *miR-132/212* loss-of-function on a 129/Sv background evoked a marked increase of light pulse-induced behavioral phase delays (Fig 4A, 4C and 4E). In contrast, we found no significant effect on phase-shifting behavior in response to a delaying light pulse in KO mice of C57BL/6N background (Fig 4B, 4D and 4E).

### Increased phase shifts in miR-132/212-deficient 129/Sv mice are accompanied by diminished light induction of Per gene transcription in the SCN

**Fig 5:**
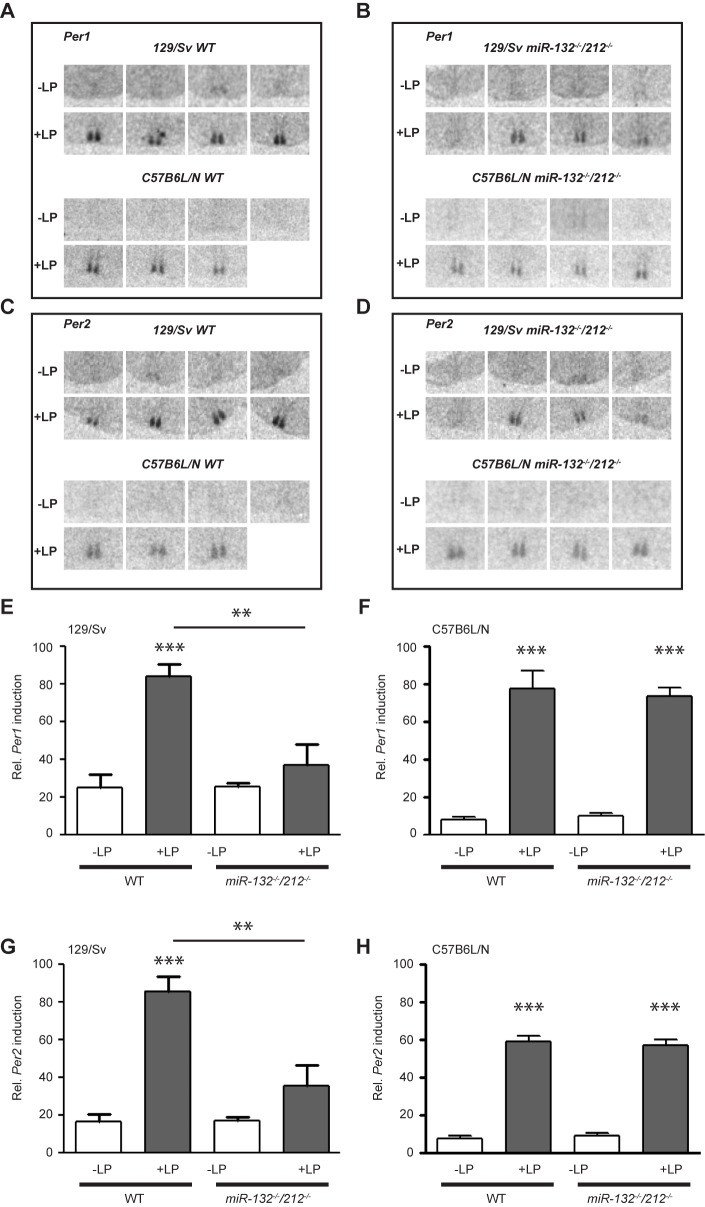
Light induced Per gene expression. Light induced *Per1* (A, B) and *Per* 2 expression (C, D) in the SCN of individual WT (A, C) and KO (B, D) mice in 129/Sv and C57Bl/6N backgrounds after a light pulse (100 lx, 1 h) at CT15 as shown by *in situ* hybridization. (E-H) Quantification of *Per1* (E, F) and *Per2* (G, H) mRNA levels in the SCN of WT or KO mice measured by radio-labelled ISH. *Per1*: 129/Sv: -LP: 25.2 +/- 7; +LP: 83.8 +/- 6.6; KO: -LP: 25.8 +/- 6.6; +LP: 25.8 +/- 2.0; C57BL/6N: -LP: 8.2 +/- 1.1; +LP: 77.5 +/- 9.4; KO: -LP: 9.8 +/- 1.5; +LP: 73.5 +/- 4.8, *Per2*: 129/Sv: -LP: 16.7 +/- 3.7; +LP: 85.8 +/- 7.6; KO: -LP: 17.1 +/- 2.1; +LP: 35.5 +/- 11.3; C57BL/6N: -LP: 8.9 +/- 1.3; +LP: 76.0 +/- 11.3; KO: -LP: 10.9 +/- 1.7; +LP: 80.2 +/- 5.3; n = 4. 129/Sv: -LP *vs*. +LP, two-way ANOV for interaction *Per1*: F(1. 12) = 10.48, p = 0.0071, Sidak’s post-hoc test for light conditions: WT: p < 0.001; KO: p > 0.05, Sidak’s post-hoc test for genotype: -LP: p > 0.05, +LP: p < 0.01; *Per2*: F(1. 12) = 12.62, p = 0.004, Sidak’s post-hoc test for light conditions: WT: p < 0.001; KO: p > 0.05, Sidak’s post-hoc test for genotype: -LP: p > 0.05, +LP: p < 0.01. C57BL/6N: -LP *vs*. +LP, two-way ANOVA for interaction *Per1*: F(1.11) = 0.4042, p = 0.5379; two-way ANOVA for light conditions F(1. 11) = 209.6, p < 0.0001; *Per2*: two-way ANOVA for interaction F(1.12) = 0.0327, p = 0.8595; two-way ANOVA for light conditions F(1. 12) = 116.4, p < 0.0001. Data are represented as mean ± SEM, ** p < 0.01, *** p < 0.001.

Photic phase shifting involves acute light induction of the core clock genes *Per1* and *Per2* in the SCN [6]. It has been reported that, *miR-132* transcription itself is also light inducible and *miR-132* induction inhibits light induced *Per1* and *Per2* expression [28]. To test whether loss-of-function of *miR-132/212* affects light induced *Per* transactivation in different mouse lines, we examined photic induction of *Per1* and *Per2* in response to a 15-min light pulse (20 lux) at CT15 in both genotypes and strains by *in situ* hybridization on SCN-containing sections. In line with the literature [6], *Per1* and *Per2* mRNA levels were strongly induced by light exposure in the SCN of WT mice of both background strains (Fig 5A, 5C and 5E–5H). Interestingly, *miR-132/212-*deficiency abolished elevation of *Per1* and *Per2* mRNA levels after photic stimulation in 129/Sv mice, even though behavioral phase delays were increased (compare Fig 4A, 4C and4E and Fig 5A–5D, 5E and 5G). In contrast, similar *Per1* and *Per2* induction was found in C57BL/6N WT and KO mice (Fig 5A–5D, 5F and 5H). This is consistent with a similar trend in behavioral resetting observed in WT and KO mice following a phase-delaying light pulse on this background (Fig 4B, 4D and 4E).

## Discussion

Our work highlights strain-specific functions of *miR-132/212* in the regulation of the murine SCN pacemaker. Our data confirm previous experiments using *miR-132* intra-cerebral antagomir infusion and overexpression in the forebrain showing that *miR-132* acts as a negative modulator of light-induced resetting of the SCN pacemaker [28, 29]. Moreover, we show that this effect is restricted to the 129/Sv background and absent in C57BL/6N mice. Our results further suggest a strain and light-regimen specific function of *miR-132/212* in the clock machinery itself, as revealed by strain-dependent effects on the free-running period length in DD and LL. These results are summarized in Table 1.

**Table 1:** Circadian analyses summary.

Reference	present study		[29]	[28]
Treatment	Knockout (*miR-132*^*-/-*^*/212*^*-/-*^)		Overexpression (*miR-132*)	Knockdown (*miR-132*)
**Strain**	129S2/SvPasOrlRj	C57BL/6N (back-crossed from 129/Sv for > 6 generations)	C57BL/6J (back-crossed from FVB/N for ≥ 4 generations)	C57BL/6J
**Rhythmicity (SCN)** *miR-132*: *miR-212*:	rhythmic rhythmic	n.d. n.d.	n.d. n.d.	rhythmic
**Peak / Nadir**	CT11 / CT23	n.d.	n.d.	~CT6 / CT19
**Period (DD)**	longer	no effect	n.d.	no effect
**Period (LL)**	no effect	longer	n.d.	n.d.
**Phase shift ZT/CT14**	increased	no effect	decreased	increased
**Light induction Per1**	abolished *Per1* induction	no effect	reduced PER1 induction	reduced *Per1* induction
**Light induction Per2**	abolished *Per2* induction	no effect	reduced PER2 induction	increased PER2 induction compared to vehicle (but below baseline)

n.d. not determined.

We found the clock modulator *miR-132* and *miR-212* to be expressed rhythmically in the central circadian clock. *miR-212* and *miR-132* share the same seed region and they can therefore target the same mRNAs [27]. Indeed, double-targeting of both *miRs* was demonstrated for mRNAs [40] implicated e.g. in circadian functions [41].

### miR-132/212 regulate core clock properties in a background-dependent manner

We found that *miR-132/212*-deficiency affects SCN clock properties as manifested by lengthening of the free-running period length in both inbred strains examined. Interestingly, neither overexpression nor knockdown of *miR-132* had an effect on the core clock mechanism (results from different studies are compared in Table 1). The *miR-132/212* gene knockout used in our study results in a complete loss-of-function whereas a knockdown approach might not be as efficient. Our double knockout is also efficiently circumventing a potential *miR-132* and *miR-212* redundancy. *miR-132* and *miR-212* show overlapping circadian oscillations in the SCN with slightly different phases. Although they are splice variants from a single locus and thus, likely act synergistically, it is possible that genetic interaction of both *miR*s could account for the period effects missed in previous studies [28, 29].

Importantly, depending on light conditions, longer periods were found in KO mice of both genetic backgrounds. Such period changes indicate that *miR-132/212-*deficiency significantly impairs the properties of the endogenous circadian clockwork in both strains. Recall that *Per1* mutant mice show a longer period under constant light exposure [42] and period lengthening in constant light has been reported when degradation of PER2 in the SCN is delayed [43]. Modulation of *Per* gene activity in the SCN of *miR-212/132*^-/-^ mice may affect the ability of the central clock to respond to light. Importantly, it has been reported that *miR-132* target genes *Poly(A) binding protein interacting protein 2* (*Paip2a)* and *B-cell translocation gene 2*, *anti-proliferative* (*Btg2*) dampen basal *Per* expression and attenuate PER protein translation, and thereby accelerate the inhibition of *Per* gene transcription [29, 44]. Thus, *miR-132/212-*deficiency may enhance the free-running period in *miR-132*^*-/-*^*/212*^*-/-*^ mice via this mechanism. Nevertheless, we cannot exclude that some of the observed effects might be mediated by miR effects in SCN-afferent tissues such as the retina where *miR-132/212* expression has been found [45] or the bed nucleus of the stria terminalis. Furthermore, baseline differences of clock gene expression in miR-deficient mice between both strains can also influence the circadian phenotype including SCN light responsiveness.

### miR-132/212 regulates phase shifts in a background-dependent manner

We tested the prediction that *miR-132/212*-deficiency influences light sensitivity of the SCN by measuring photic phase shifts of locomotor behavior [46]. Indeed, we found that *miR-132/212*-deficiency in 129/Sv mice modulates circadian light responses, with respect to resetting and *Per* gene induction. Additionally, a prolonged period length during constant light as observed in KO C57BL/6N mice further suggests an increased light sensitivity of the SCN [39]. Phase shifting of the circadian clock in response to light stimulation is believed to be mediated by *Per* induction in the SCN which then triggers a resetting of behavioral activity [6, 47]. In accordance, miR-132 overexpression in C57BL/6 mice resulted in reduction of light-induced PER1/2 levels and photic resetting of behavior [29]. We found that *miR-132*^*-/-*^*/212*^*-/-*^ C57BL/6N mice did not exhibit a change in *Per* expression upon light stimulation, which is consistent with their unaffected behavioral resetting. Interestingly, although light responsiveness of *Per1* and *Per2* expression in the SCN was completely abolished in *miR-132*^*-/-*^*/212*^*-/-*^ 129/Sv mice, they demonstrated enhanced phase-shifting behavior. This is in accordance with previous reports using *miR-132* antagomirs in C57BL/6J mice which led to a marked reduction of light induced *Per1* gene expression and enhanced phase shifts [28]. Nevertheless, loss-of-function of, both, *miR-132* and *miR-212* on the 129/Sv background seemed to further enhance the previously described circadian resetting phenotype. Of note, *Per1*-deficient mice exhibit an increased phase shifting capacity similar to the one documented in *miR-132*^*-/-*^*/212*^*-/-*^ 129/Sv mice [48]. However, other mechanisms than *Per* regulation may play a role in determining resetting in *miR-132*^*-/-*^*/212*^*-/-*^ mice, e.g. it can be speculated that the weaker adaptation ability of *miR-132*^*-/-*^*/212*^*-/-*^ mice, similar to *Per1-*deficient mice, may be the result of a weakened circadian oscillator, indicated by reduced circadian pacemaker amplitude, as was also suggested for *Clock* mutant mice [49].

Contradictory results between knockout, knockdown and overexpression studies (summarized in Table 1) might be explained by parallel, but independent effects of *miR-132* and *miR-212* on behavioral resetting and *Per* gene expression in the SCN.

Direct targets of *miR-212* and *miR-132* in the SCN are known to interact with *Per* genes [29, 44]. In particular, *Methyl-CpG-binding protein 2 Mecp2*, which is involved in chromatin remodeling and a target of *miR-132* and *miR-212*, activates *Per* transcription while the *miR-132* target genes PAIP2A and BTG2 attenuate PER protein translation, both of which may result in a dampening of light induced *Per* expression or accelerated PER decay [29, 44], thus, influencing behavioral resetting. This link between *Per* gene modulation and phase shift regulation by the *miR-132/212* family, however, needs to be further examined.

Circadian phenotypes in *miR-132*^*-/-*^*/212*^*-/-*^ mice were generally more pronounced in the 129/Sv than in the C57BL/6N strain. These data support the possibility that different mouse strains may carry different genetic modifiers influencing the circadian phenotype. Similarly, a *Clock*^Δ19^ modulator gene has recently been identified which accounts for the suppression of the *Clock*^Δ19^ mutant circadian phenotype in BALB/cJ mice [50]. Interestingly, *miR*s act as modifiers [19] and impact on circadian clock function [28] and covariation in *miR-212* abundance and function was documented in different background strains, including C57BL/6 [17, 18, 51].

Taken together, we have shown that *miR-132/212* are background-dependent modulators of the circadian clock. Future studies are required to identify the *miR132/212* targets whose regulation may mediate this effect. Our data further emphasize that, to fully understand the regulatory function of a specific gene or a *miR*, loss-of-function phenotypes should be analyzed in more than one genetic background strain. The persistence of a phenotype across various strains would underscore a universal function of a specific gene and, thus, suggest a potential significance for humans.
